# MdWRKY11 improves copper tolerance by directly promoting the expression of the copper transporter gene *MdHMA5*

**DOI:** 10.1038/s41438-020-0326-0

**Published:** 2020-07-01

**Authors:** Kun Shi, Xuan Liu, Yunpeng Zhu, Yixue Bai, Dongqian Shan, Xiaodong Zheng, Lin Wang, Haixia Zhang, Chanyu Wang, Tianci Yan, Fangfang Zhou, Zehui Hu, Yanzhao Sun, Yan Guo, Jin Kong

**Affiliations:** 1 College of Horticulture, China Agricultural University, 100193 Beijing, China; 2 College of Biological Sciences, China Agricultural University, 100193 Beijing, China

**Keywords:** Plant molecular biology, Plant stress responses

## Abstract

Overuse of fungicides and fertilizers has resulted in copper (Cu) contamination of soils and toxic levels of Cu in apple fruits. To breed Cu-resistant apple (*Malus domestica*) cultivars, the underlying molecular mechanisms and key genes involved in Cu resistance must be identified. Here, we show that MdWRKY11 increases Cu tolerance by directly promoting the transcription of *MdHMA5*. MdHMA5 is a Cu transporter that may function in the storage of excess Cu in root cell walls and stems for Cu tolerance in apple. The transcription factor MdWRKY11 is highly induced by excess Cu. *MdWRKY11* overexpression in transgenic apple enhanced Cu tolerance and decreased Cu accumulation. Apple calli transformed with an *MdWRKY11*-RNAi construct exhibited the opposite phenotype. Both an in vivo chromatin immunoprecipitation assay and an in vitro electrophoretic mobility shift assay indicated that MdWRKY11 binds to the promoter of *MdHMA5*. Furthermore, MdWRKY11 promoted *MdHMA5* expression in transgenic apple plants, as revealed by quantitative PCR. Moreover, inhibition of *MdWRKY11* expression by RNA interference led to a significant decrease in *MdHMA5* transcription. Thus, MdWRKY11 directly regulates *MdHMA5* transcription. Our work resulted in the identification of a novel MdWRKY11-MdHMA5 pathway that mediates Cu resistance in apple.

## Introduction

Apple (*Malus domestica*) is one of the four most widely cultivated fruit crop species, and ensuring that apples do not accumulate toxic levels of metals from the soil is important for public health^1,2^. The widespread use of the Bordeaux fungicide mixture, farmyard manure containing Cu as fertilizer, and wastewater for irrigation has led to the accumulation of excess Cu in the soil and in apple fruits^1^. Indeed, the Cu levels of apples in many orchards have been reported to be ten times higher than safe limits^3,4^, and the problem is getting worse. The threat to human health from toxic, Cu-contaminated apple fruits is a long-term problem because it removing excess Cu already present in soils is challenging^5^.

An extreme excess amount of Cu in the soil leads to leaf chlorosis, limits apple tree growth, and greatly reduces yield^6^; however, light-to-moderate Cu pollution of orchard soils, which does not cause these symptoms, probably poses a greater threat to human health, as toxic, Cu-contaminated apple fruits can continue to be produced by trees that show no signs of Cu stress, causing the problem to go undetected^1,7^. Therefore, it is important to elucidate the molecular mechanisms underlying the response to excess Cu in apple both for monitoring Cu contamination and for molecular breeding of Cu-resistant apple cultivars.

Excess Cu inhibits photosystem II activity and photosynthesis, impairs the elongation of roots and shoots, reduces fruit quality and yield, and can trigger senescence and death^8–11^. To withstand excess Cu in the soil, plants have developed two strategies for maintaining normal Cu levels in their tissues: Cu efflux and Cu sequestration^12^. When excess Cu enters root epidermal cells, the first strategy is to export Cu back outside the plant cytoplasm, possibly via storage in the root cell wall^12,13^. The second strategy is to store excess Cu in tissues that are less sensitive to the toxic effects of Cu, such as stem tissue^12^. In this second case, in chelated form, Cu moves up the stem through the transpiration stream after being transported into xylem^14^. Chelation not only decreases the cytosolic free Cu concentration, thereby reducing photosystem II damage, but also facilitates Cu transport through the plant^15^. Once free Cu is chelated by metallothionein or phytochelatin proteins, metallochaperones deliver the Cu–ligand complexes directly to P_1B_-type ATPases for transport^16,17^.

Heavy Metal ATPase 5 (HMA5) is a Cu-specific P_1B_-type ATPase that transports chelated Cu across membranes^18^. HMA5 is involved in Cu tolerance in two ways: it transports Cu out of the root, and it mediates Cu uploading for long-distance transport and redistribution within the plant. In the Cu export strategy, HMA5 in the plasma membrane of root epidermal cells transports excess Cu out of the cytoplasm to maintain proper Cu levels in the plant^19^. AtHMA5, which has been identified as component of a QTL in *Arabidopsis thaliana*, transports chelated Cu outside root epidermal cells under Cu excess stress^20,21^. The Arabidopsis *hma5* mutant is hypersensitive to excess Cu and accumulates relatively large amounts of Cu in its roots^16^. A similar function was reported for SvHMA5II in *Silene vulgaris*^21^. With respect to the redistribution strategy, chelated Cu moves laterally from cell to cell via HMA5 transporters and is ultimately uploaded to the xylem for transport from the roots to the stem^22,23^. The Cu insensitivity of the stem makes this tissue an ideal place to sequester excess Cu away from Cu-sensitive organs such as roots and leaves^19,23^. In rice (*Oryza sativa*), OsHMA5 is localized in the plasma membrane of root pericycle cells, where it loads Cu into the xylem for long-distance transport to stems^22,24^. However, there have not been any reports of *HMA5* genes in woody plant species, in which the Cu resistance mechanism is expected to be even more complex.

Transcription factors (TFs) play a central role in the response to excess heavy metal by orchestrating several physiological processes^25–28^. There have been several reports on the transcriptional regulation of the Cu response in multicellular eukaryotes. The transcription factors SPL7, CRR1, and Ace1-like protein regulate the Cu chaperones *CCH* (involved in Cu chelation and detoxification), *COPT1* (involved in Cu absorption), and *FeSOD* and *Cu/ZnSOD* (involved in reactive oxygen species mitigation), respectively^29–31^. However, the transcriptional regulation of *HMA5* under excess Cu remains unknown.

WRKY TFs play a critical role in the response to excess heavy metals (iron, cadmium, and aluminum) by regulating their chelation and translocation of the metals and by reducing secondary oxidative damage^32–34^. WRKYs belong to one of the largest TF families in plants and are named for their highly conserved WRKYGQK heptapeptide at the N-terminus, which specifically binds to W-box *cis*-elements (containing a TTGACC/T core sequence) in the promoters of downstream target genes^35–37^. However, it is not known whether WRKY TFs are involved in the response to excess Cu or what regulatory pathways might be involved.

In this study, we isolated *MdWRKY11*, which is significantly induced in response to Cu stress, in apple. Overexpression of *MdWRKY11* conferred increased Cu tolerance to transgenic apple trees. Furthermore, we demonstrated that MdWRKY11 directly binds to the promoter of *MdHMA5*, which encodes a P_1B_ ATPase, and activates its expression. MdHMA5 functions in Cu transport and decreases Cu accumulation in apple plants. In addition to isolating a novel transcriptional regulatory pathway of Cu tolerance in plants, this study provides marker genes for monitoring Cu contamination in orchards.

## Results

### Expression of *MdWRKY11* in response to CuSO_4_ treatment

**Fig. 1:**
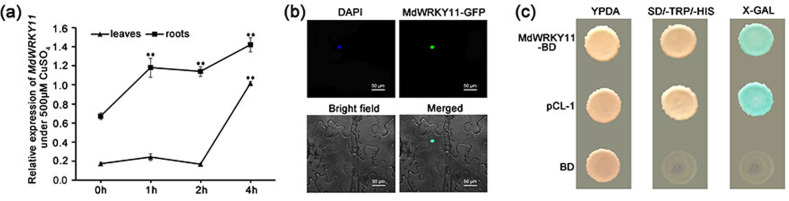
Expression, subcellular localization, and transcriptional activity of MdWRKY11. **a***MdWRKY11* expression in the leaves and roots under excess Cu stress, as detected by qPCR. The *MdWRKY11* expression level was normalized to the internal *MdActin* expression level. The apple plants were treated with 500μM CuSO_4_ for 0, 1, 2, and 4h. The data are the means±SDs of triplicate experiments for each time point. The asterisks indicate values that are significantly different from those of the control (Student’s *t*-test): *P < 0.05; **P < 0.01. **b** MdWRKY11-GFP is localized to the nucleus of *Nicotiana benthamiana* cells. *35S::MdWRKY11-GFP* was transiently expressed in epidermal cells of *N. benthamiana* leaves and visualized by confocal microscopy (×40). The nucleus was dyed with 4,6-diamidino-2-phenylindole (DAPI). **c** Transcriptional activation of MdWRKY11 in yeast cells. Yeast AH109 strains expressing *pCL-1*, binding domain (*BD*), and *pBD-MdWRKY11* were cultured on yeast peptone dextrose adenine agar (YPDA) or selective SD-His-Trp media. pCL-1 encoding the GAL4 protein and the empty vector pGBKT7 (BD) were used as the positive and negative controls, respectively

To identify *WRKY* genes that might be involved in the response to excess Cu, we screened the expression of 29 candidate *MdWRKY*s in the leaves and roots of hydroponic plants treated with 500 μM CuSO_4_. Among these *MdWRKY*s, *MdWRKY11* expression was significantly induced in response to CuSO_4_ treatment in both the roots and the leaves (Fig. 1a), suggesting that this gene has an important role in the response to excess Cu. Therefore, we selected *MdWRKY11* for further study.

### Subcellular localization of MdWRKY11

To examine the subcellular localization of MdWRKY11, *35S::MdWRKY11-GFP* was infiltrated into *N. benthamiana* leaves via *Agrobacterium*-mediated transient transformation. The MdWRKY11-GFP fluorescence was localized exclusively to the nucleus (Fig. 1b).

### Transcriptional activity of MdWRKY11

The transcriptional activation activity of MdWRKY11 was assayed in a yeast system. Yeast cells transformed with *pBD-MdWRKY11* or the positive control construct pCL-1 grew well on SD-Trp-His selective media and displayed α-galactosidase activity, whereas yeast cells carrying the negative control construct pGBKT7 were unable to grow on the selective medium (Fig. 1c). These results indicate that MdWRKY11 is a transcriptional activator in the yeast system.

### Cu tolerance of transgenic apple plants overexpressing *MdWRKY11*

To investigate the potential function of *MdWRKY11* in Cu tolerance, transgenic apple plants overexpressing *MdWRKY11* were generated via *Agrobacterium*-mediated transformation. The expression of *MdWRKY11* in OEWRKY11-1, OEWRKY11-2, and OEWRKY11-3 transgenic apple lines was significantly higher than that in the untransformed controls (Fig. S1a). Therefore, we selected these three lines for further analysis.

**Fig. 2:**
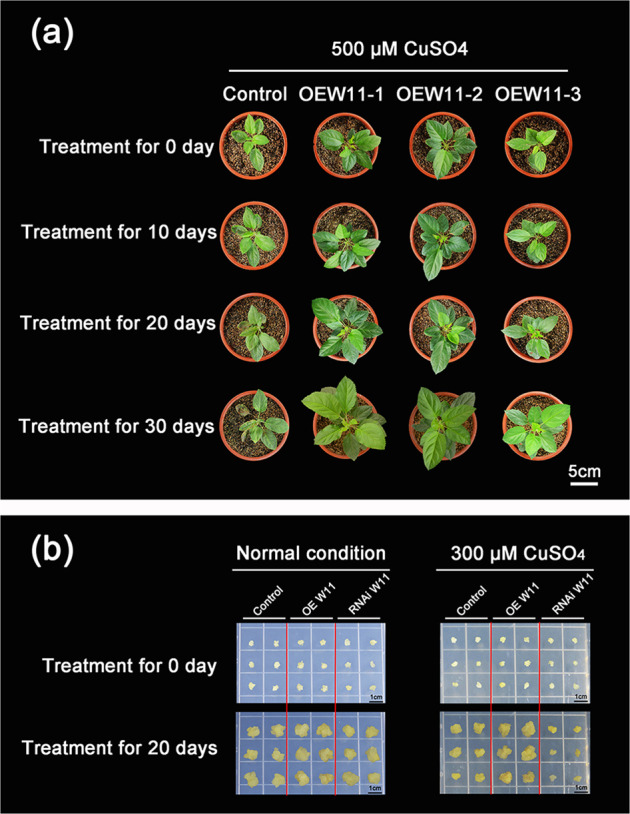
Assessment of Cu tolerance of transgenic apple plants and calli subjected to CuSO_4_ treatment. **a** Phenotypes of three transgenic apple lines overexpressing *MdWRKY11* and an untransformed control plant treated with 500μM CuSO_4_ for 10, 20, and 30 days. **b** Cu tolerance of transgenic *MdWRKY11*-overexpressing and *MdWRKY11* RNAi calli and control calli cultured on media supplemented with excess Cu (300μM CuSO_4_) or normal Cu concentrations for 20 days

The control apple plants grew slowly under excess Cu conditions. After thirty days of Cu treatment, the older leaves displayed chlorosis and brown spots, and the newer leaves turned yellow. However, these toxic symptoms were not observed in the transgenic plants (Fig. 2a). Therefore, the overexpression of *MdWRKY11* conferred enhanced Cu tolerance to the transgenic apple plants.

We also examined *MdWRKY11* expression and Cu tolerance in transgenic apple calli harboring either the overexpression construct or an *MdWRKY11* RNA interference construct. *MdWRKY11* overexpression or underexpression was confirmed by qPCR (Fig. S1b). Similar to that which occurred for the plants transformed with the overexpression construct, transgenic apple calli overexpressing *MdWRKY11* presented enhanced Cu tolerance. Calli in which *MdWRKY11* expression had been decreased by the RNAi construct presented decreased Cu tolerance (Fig. 2b). Under normal conditions, the control calli and both types of transgenic calli appeared to grow at similar rates. In the presence of CuSO_4_, however, calli overexpressing *MdWRKY11* grew better than the control, whereas calli carrying the RNAi construct grew more slowly. Overall, *MdWRKY11* overexpression resulted in increased Cu tolerance, while decreased *MdWRKY11* expression resulted in decreased Cu tolerance.

### Effects of *MdWRKY11* overexpression on Cu accumulation in the roots and leaves of transgenic apple plants

**Fig. 3:**
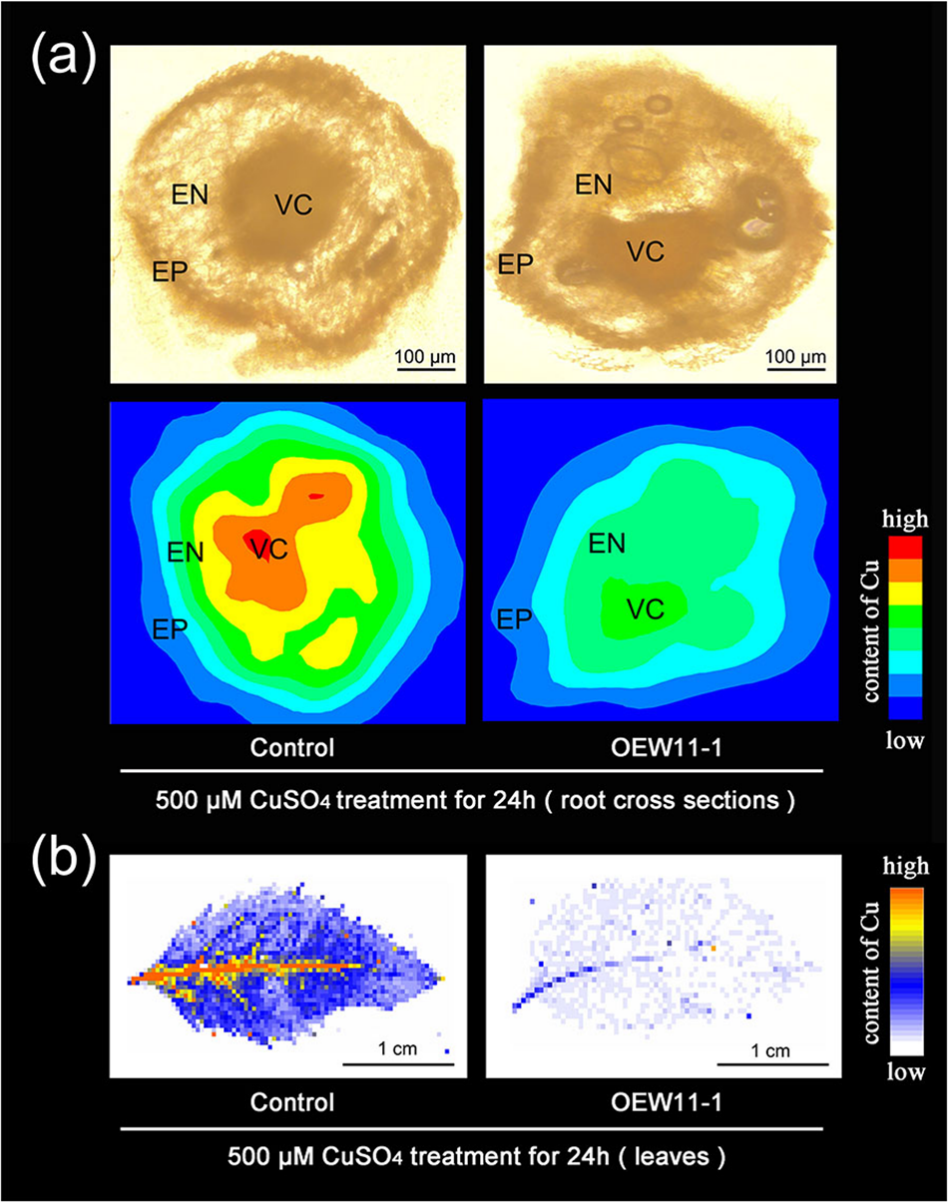
Cu levels and distribution shown as μ-XRF elemental maps of the roots and leaves of transgenic plants overexpressing *MdWRKY11* and an untransformed control plant treated with 500 μM CuSO_4_ for 24 h. **a** Cu level and distribution depicted as μ-XRF elemental maps of the roots. VC vascular cylinder, EN endodermis, EP epidermis. **b** Cu level and distribution shown as μ-XRF elemental maps of leaves

To further investigate the role of *MdWRKY11* in Cu tolerance, we used X-ray fluorescence (XRF) microtomography to analyze the content and distribution of Cu in control plants and *MdWRKY11*-overexpressing plants treated with excess Cu. The same pattern of Cu distribution was observed in both the control and transgenic apple plants. The highest Cu level was in the vascular cylinder (VC). The Cu level decreased with increasing distance from the VC, being highest in the endodermis (EN) and lowest in the epidermis (EP). Consistent with their Cu-tolerant phenotype, the *MdWRKY11*-overexpressing plants had significantly less Cu than did the control plants in their roots and leaves (Fig. 3).

### Binding of MdWRKY11 to the *MdHMA5* promoter and its effect on *MdHMA5* transcription

**Fig. 4:**
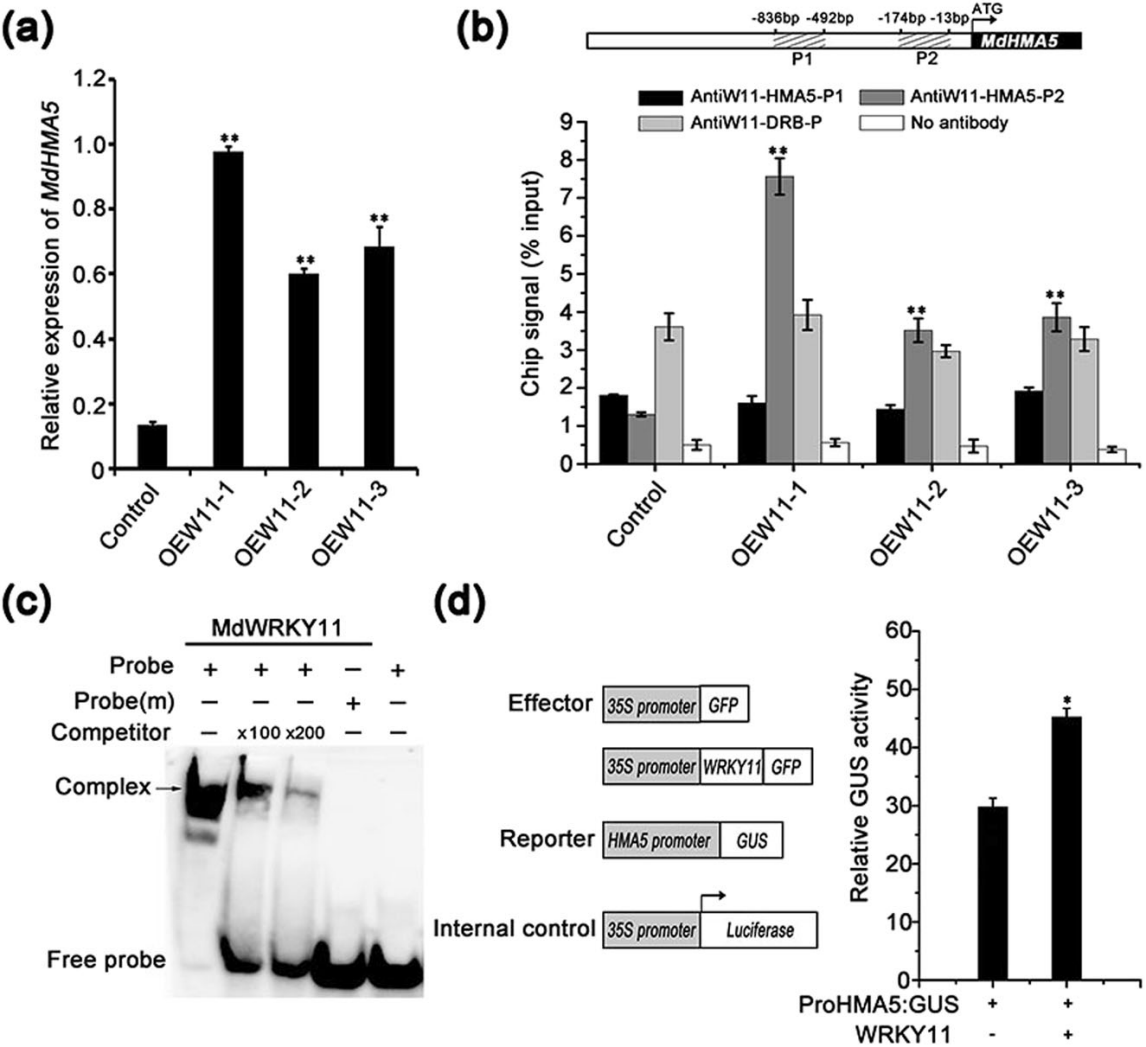
MdWRKY11 binds to the promoter and activates the expression of *MdHMA5*, which encodes a Cu-transporting P_1B_-type ATPase. **a***MdHMA5* expression in transgenic and control apple plants. **b** Binding of MdWRKY11 to the W-box of the *MdHMA5* promoter indicated by chromatin immunoprecipitation (ChIP)-qPCR. The position of the W-box is indicated by the gray bar. The ChIP signal was quantified as the percentage of immunoprecipitated DNA out of the total input DNA, as determined by qPCR. A parallel experiment without antibodies and the promoter of *MdDRB* served as the negative control. **c** Electrophoretic mobility shift assay (EMSA) results confirming the in vitro binding of MdWRKY11 to the *MdHMA5* promoter fragment. The arrow indicates the position of a protein–DNA complex after incubation with GST-MdWRKY11 and the biotin-labeled DNA probe *MdHMA5*. Both the probe containing a W-box and the probe (m) containing a mutated W-box were synthesized according to the sequence of the *MdHMA5* promoter. **d** Relative GUS activity normalized with respect to luciferase (LUC) activity in transiently transformed apple calli expressing *35S::MdWRKY11-GFP*, *proMdHMA5::GUS*, and *35S::LUC*; the relative GUS activity in transiently transformed apple calli expressing *35S::GFP*, *proMdHMA5::GUS*, and *35S::LUC* served as the control. The data are the means±SDs of triplicate experiments. The asterisks indicate values that are significantly different from those of the control (Student’s *t*-test): *P < 0.05; **P < 0.01

To determine how *MdWRKY11* overexpression provides increased Cu tolerance, we analyzed the expression of key genes involved in Cu absorption and transport (Fig. 4a and S2). Among these genes, the expression of *MdHMA5*, which encodes a Cu-specific transporter, was significantly higher in the *MdWRKY11*-overexpressing lines than in untransformed control plants (Fig. 4a). Similarly, *MdHMA5* expression in transgenic calli overexpressing *MdWRKY11* was nearly 50% higher than that in control calli. Conversely, *MdHMA5* expression decreased to nearly half the control levels in calli transformed with the *MdWRKY11* RNAi construct (Fig. S4a). These results suggest that MdWRKY11 positively regulates the expression of *MdHMA5*.

To test whether *MdHMA5* is directly regulated by MdWRKY11, we examined whether MdWRKY11 binds to the *MdHMA5* promoter both in vivo and in vitro using chromatin immunoprecipitation (ChIP)-qPCR and EMSAs, respectively. ChIP-qPCR analysis showed that the P2 fragment, which contains the W-box motif of the *MdHMA5* promoter, was enriched in samples from the transgenic lines (Fig. 4b), confirming that MdWRKY11 binds specifically to the *MdHMA5* promoter in vivo. EMSAs demonstrated the binding of MdWRKY11 to the P2 fragment of the *MdHMA5* promoter in vitro. This binding was reduced in a dose-dependent manner with the addition of a 100-fold or 200-fold excess of unlabeled competitor. In addition, the binding was completely abolished when the probe contained a mutated W-box element, further confirming that the W-box of the P2 fragment of the *MdHMA5* promoter is the binding site for MdWRKY11 (Fig. 4c).

We further tested the effects of MdWRKY11 on *MdHMA5* expression in apple calli transiently cotransformed with an *MdWRKY11* overexpression construct and with a construct in which *GUS* expression was driven by the *MdHMA5* promoter. The ratio of GUS to LUC activity was significantly higher in calli expressing *proMdHMA5::GUS*, *35S::MdWRKY11-GFP*, and *35S::LUC* than in control calli without the *35S::MdWRKY11-GFP* construct (Fig. 4d). Taken together, these results indicate that MdWRKY11 specifically binds to the *MdHMA5* promoter and activates its expression.

### Phylogenetic analysis, subcellular localization, and functional analysis of MdHMA5

**Fig. 5:**
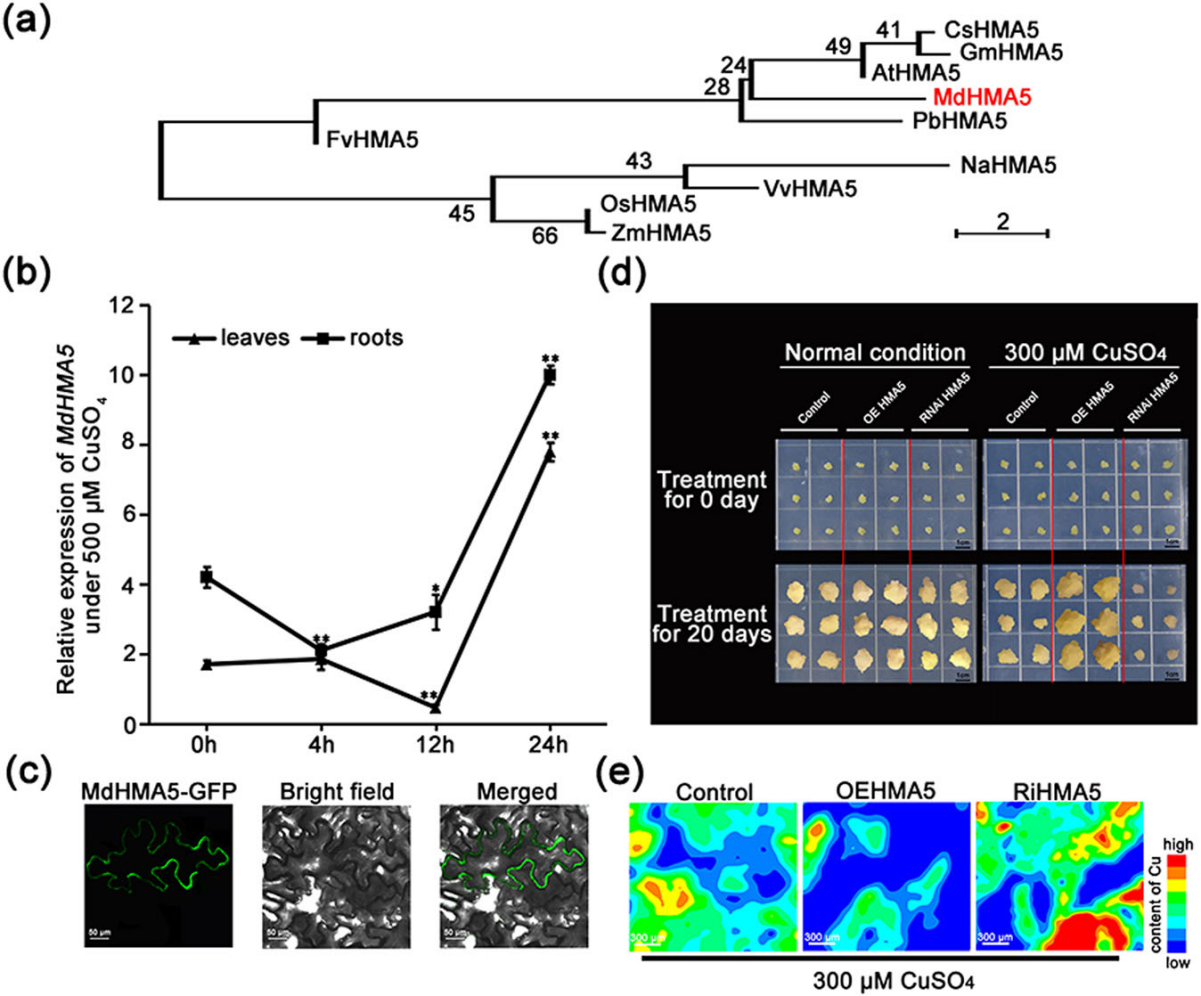
Phylogenetic analysis, subcellular localization, and functional analysis of MdHMA5. **a** Phylogenetic tree of the HMA5 subgroup of heavy metal ATPases from select plant species. The species and the accession numbers of the amino acid sequences used in the analysis are as follows: *Arabidopsis thaliana* (AT1G63440), *Oryza sativa* (OS04G46940), *Fragaria vesca* (FvH4_5g02091), *Vitis vinifera* (XP_010651259.1), *Cucumis sativus* (CSPI05G05530), *Zea mays* (ZM2G143512), *Nicotiana attenuata* (XP_019241377.1), *Glycine max* (GM11G024400), and *Pyrus* x *bretschneideri* (XP_018506630.1). **b***MdHMA5* expression in the leaves and roots under excess Cu treatment measured by qPCR and normalized according to the *MdActin* expression level. Hydroponic apple plants under excess Cu stress were sampled at 0, 4, 12, and 24h of treatment. The data are the means±SDs of triplicate experiments for each time point. The asterisks indicate the values that are significantly different from the controls (Student’s *t*-test): *P < 0.05; **P < 0.01. **c** MdHMA5-GFP is localized to the plasma membrane of *Nicotiana benthamiana* leaf epidermal cells. *35S::MdHMA5-GFP* was transiently expressed in epidermal cells of *N. benthamiana* leaves and visualized by confocal microscopy (×40). **d** Cu tolerance of transgenic *MdHMA5*-overexpressing and *MdHMA5-*RNAi calli and untransformed control calli treated with excess Cu (300μM) for 20 days. **e** Cu levels, shown as μ-XRF elemental maps, in transgenic *MdHMA5*-overexpressing and *MdHMA5-*RNAi calli and control calli under excess Cu stress for 20 days

Phylogenetic analysis showed that MdHMA5 is more closely related to AtHMA5 (Fig. 5a), which plays an important role in Cu homeostasis and detoxification in Arabidopsis^16,21^, than to other HMA5s from other species. *MdHMA5* expression was significantly induced by excess CuSO_4_ treatment in both the roots and leaves of hydroponic plants (Fig. 5b). Similar to AtHMA5, MdHMA5 was localized to the plasma membrane^16,21^ (Fig. 5c). These results suggest that, like AtHMA5, MdHMA5 may be involved in the detoxification of excess Cu.

To investigate the function of *MdHMA5* in Cu detoxification, apple calli were transformed with *MdHMA5* overexpression or *MdHMA5-*RNAi constructs and cultured on media containing normal or excess amounts of Cu (Fig. 5d). All of the calli grew well under normal conditions, with no significant differences. On media supplemented with excess Cu, however, the *MdHMA5*-overexpressing calli grew markedly better than did the controls, while the growth of the *MdHMA5-*RNAi calli was more severely inhibited. Furthermore, XRF microtomography analysis showed that the highest Cu level occurred in *MdHMA5-*RNAi calli and that the lowest Cu level occurred in the *MdHMA5*-overexpressing calli (Fig. 5e). These results indicate that the overexpression of *MdHMA5* confers increased Cu tolerance to apple calli by maintaining the Cu level, while *MdHMA5*-RNAi calli exhibited the opposite phenotype. These results are in good agreement with the results of our analysis of transgenic apple plants overexpressing *MdWRKY11*.

## Discussion

Cu contamination has become a severe problem in apple orchards by impairing growth and reducing apple yield. Importantly, human health may be threatened by toxic apple fruit production^1^. Understanding the mechanisms underlying Cu resistance in apple is the basis for molecular breeding of Cu-resistant apple cultivars.

We screened apple *WRKY* genes to isolate the key transcription factor involved in Cu resistance. *MdWRKY11* expression was significantly induced by excess Cu (Fig. 1a) and was chosen for subsequent research. MdWRKY11 is a typical Group II WRKY transcription factor; it is located in the nucleus and functions as a transcriptional activator in a yeast system (Fig. 1b, c). We overexpressed *MdWRKY11* in Gala, a popular apple cultivar grown worldwide^38^, which conferred increased Cu tolerance to the transgenic plants. Moreover, a similar Cu-tolerant phenotype was observed for transgenic apple calli overexpressing *MdWRKY11*, while calli transformed with an *MdWRKY11* RNAi construct were less tolerant to excess Cu (Fig. 2b). These results imply that MdWRKY11 plays an important role in Cu resistance and constitute the first line of evidence that WRKY TFs are involved in regulating the response of apple to excess Cu.

To isolate the genes regulated by MdWRKY11 as part of the Cu response, we measured the expression of key genes involved in Cu absorption and transport in control plants and *MdWRKY11*-overexpressing apple plants. *MdHMA5* expression significantly increased in the plants overexpressing *MdWRKY11* (Fig. 4a). The direct regulation of *MdHMA5* was indicated by in vivo ChIP-qPCR (Fig. 4b), by in vitro EMSAs (Fig. 4c) and by additional in vivo transgenic tests (Fig. 4d). In addition, *MdHMA5* expression was relatively low in calli transformed with an *MdWRKY11* RNAi construct (Fig. S2a), suggesting that MdWRKY11 plays a critical role in regulating the expression of *MdHMA5*. Taken together, these results demonstrate that MdWRKY11 binds to the *MdHMA5* promoter to activate its transcription under excess Cu stress.

Given that the role of HMA5 in woody plant species is not known, we investigated its involvement in Cu detoxification in apple calli transformed with *MdHMA5*-overexpression or *MdHMA5*-RNAi constructs. Under excess Cu conditions, overexpression of *MdHMA5* provided enhanced Cu tolerance, while RNAi of *MdHMA5* expression decreased Cu tolerance (Fig. 5d). This confirmed the important role of MdHMA5 in Cu detoxification.

To clarify how the MdWRKY11-MdHMA5 pathway functions in Cu resistance, we used XRF to analyze the concentration and distribution of Cu in transgenic apple plants and calli presenting altered *MdWRKY11* or *MdHMA5* expression (Fig. 5e). Under excess Cu conditions, transgenic apple plants overexpressing *MdWRKY11* had markedly lower concentrations of Cu in both their roots and leaves compared with those of the controls (Fig. 3). The Cu level of the roots was much higher than that of the leaves in both the control plants and transgenic plants (Fig. S3b, d). Compared with those of the control plants, the roots of the transgenic plants had less Cu in every tissue layer, indicating that roots of the transgenic plants accumulate less Cu than do those of the control plants, which suggests that epidermal root cells might transport Cu outside the cytoplasm more efficiently, possibly through upregulated *MdHAM5*. In root cross-sections, the Cu level was highest in the VC and decreased from the endodermis to the epidermis in both the transgenic plants and control plants. These results imply that the MdWRKY11-induced MdHMA5 pathway may also be involved in Cu loading, as has been reported in rice^24^. Indeed, physiological studies showed that, compared with Cu-sensitive apple rootstocks, Cu-tolerant apple rootstocks had lower levels of Cu in their roots, possibly the result of more efficient Cu export or redistribution^2^.

Our observations of transgenic plants suggest that apple employs a redistribution strategy to detoxify excess Cu. Furthermore, Cu levels were dramatically lower in transgenic calli overexpressing *MdHMA5* and higher in *MdHMA5*-RNAi calli, indicating that MdHMA5 is also involved in Cu export. Therefore, MdHMA5, which is directly regulated by MdWRKY11 and functions in transmembrane Cu transport, is possibly required for the extrusion or redistribution of Cu in apple. This would explain the significantly lower Cu levels in the roots and leaves of the transgenic apple plants overexpressing *MdWRKY11* compared with those of the controls. Together, our findings suggest that the use of the MdWRKY11-MdHMA5 pathway is a key strategy for Cu detoxification in apple.

**Fig. 6:**
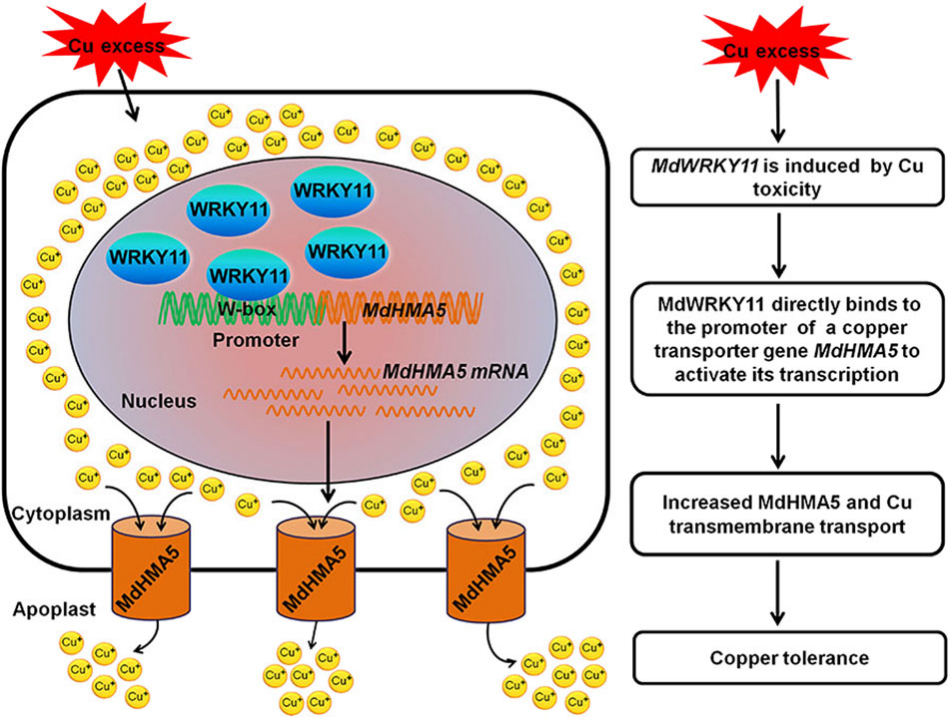
Model of the role of the MdWRKY11-MdHMA5 pathway in the response to excess Cu in apple root cells. Excess Cu-inducible MdWRKY11 binds to the promoter of *MdHMA5*, increased *MdHMA5* transcription. Increased MdHMA5 promotes transmembrane transport Cu, which more efficiently decreases Cu levels in the cytoplasm

Based on these observations, we present a model for Cu detoxification in apple (Fig. 6): excess Cu induces MdWRKY11, which directly binds to the promoter of *MdHMA5* (a Cu-transporting P_1B_-type ATPase gene), increasing its transcription. MdHMA5 then reduces Cu levels in the cytoplasm by increasing Cu transmembrane transport in root cells. Our work identified a novel MdWRKY11-MdHMA5 pathway that mediates Cu resistance in apple. This study not only contributes to the molecular breeding of Cu-resistant apple cultivars but also provides marker genes to monitor Cu contamination.

## Materials and methods

### Plant materials and growth conditions

Gala 3 (*Malus x domestica* Borkh. cv. Royal Gala) plants were cultured on MS media containing 0.2 mg l^−1^ indole-3-acetic acid (IAA), 0.3 mg l^−1^ 6-benzylaminopurine (6-BA), and 0.1 mg l^−1^ gibberellin 3 (GA3) at 23 °C under a 16 h:8 h (light/dark) photoperiod with a light intensity of 100 μmol m^−2^s^−1^, after which they were subcultured every four weeks. The Gala 3 plants were rooted and transplanted as described by Zheng et al.^39^. Apple callus induction and culture were conducted as described by Zheng et al.^39^.

### Quantitative reverse transcription PCR (qPCR)-based analysis

qPCR-based analysis was used to measure *MdWRKY11* and *MdHMA5* expression in apple plants exposed to excess Cu. Hydroponically cultivated apple plants that had 4–6 leaves and were approximately 10 cm tall were treated with Hoagland nutrient solution supplemented with 500 μM CuSO_4_. Root and leaf samples were collected at 0, 1, 2, 4, 12, and 24 h. Total RNA isolation, reverse transcription, and qPCR were performed as described by Zheng et al.^39^. *MdActin* was used as an internal control. *MdWRKY11* and *MdHMA5* expression in transgenic plants and control plants or calli was assessed similarly. All the primers used are listed in Supporting Information Table S1.

### Determination of MdWRKY11 subcellular localization

The coding sequence of *MdWRKY11* without the stop codon was amplified and subcloned into a pMDC83 vector to create a *35S::MdWRKY11-GFP* fusion construct, which was subsequently introduced into *Agrobacterium tumefaciens* strain GV3101. The primers used to amplify the construct are listed in Supporting Information Table S2. The construct was infiltrated into *N. benthamiana* leaves, and GFP fluorescence in the transgenic leaves was observed by confocal microscopy (×40) after staining with the nucleus-specific dye DAPI, as previously described by Zheng et al.^39^.

### Transcriptional activation assays in yeast

With respect to transcriptional activation assays in yeast, the *MdWRKY11* coding region without the stop codon was amplified and inserted into a pGBKT7 vector to generate a *pBD-MdWRKY11* construct for *MdWRKY11* expression as a fusion protein with the GAL4-binding domain (BD). The primers used are listed in Table S2. The transcriptional activation assay of MdWRKY11 was performed as described previously^39^. pCL-1 and pGBKT7 vectors were used as positive and negative controls, respectively.

### Generating transgenic Gala 3 apple plants and calli presenting altered *MdWRKY11* and *MdHMA5* expression

The *MdWRKY11* coding region was amplified and inserted into a pBI121 vector to generate a *35S::MdWRKY11-GUS* overexpression construct. The plasmid was then introduced into *Agrobacterium tumefaciens* strain EHA105 for subsequent *Agrobacterium*-mediated transformation of Gala3 according to the method of Dai et al.^40^. Transgenic plants were confirmed by PCR analysis, while the mRNA abundance of *MdWRKY11* in all transgenic apple lines and control plants was determined by qPCR-based analysis. Each experiment was independently repeated three times.

To generate transgenic calli overexpressing *MdWRKY11* or *MdHMA5*, the coding sequence of *MdWRKY11* or *MdHMA5* was subcloned into a pMDC83 vector to generate *35S::MdWRKY11* and *35S::MdHMA5* plasmids, respectively. To reduce *MdWRKY11* and *MdHMA5* expression in apple calli, the sense and antisense fragments of the two genes were inserted into a pZH01 RNA interference (RNAi) vector, yielding RNA interference constructs *pZH01-MdWRKY11-RNAi* and *pZH01-MdHMA5-RNAi*, respectively. The primers used in this experiment are listed in Supporting Information Table S2. The plasmids were introduced into *Agrobacterium tumefaciens* strain EHA105. Apple calli were transformed using the method described by An et al.^41^. Transgenic calli with altered expression levels of *MdWRKY11* and *MdHMA5* were confirmed by PCR-based analysis, and the expression levels of *MdWRKY11* or *MdHMA5* in all the transgenic lines and control calli were quantified via qPCR. Each experiment was independently repeated three times.

### Determination of the Cu tolerance of transgenic apple plants or calli presenting altered levels of *MdWRKY11* or *MdHMA5* expression

To determine the Cu tolerance of transgenic apple plants, transgenic apple plants overexpressing *MdWRKY11* and control plants were watered with full-strength Hoagland nutrient solution supplemented with 500 μM CuSO_4_ every three days, and the pH of the nutrient solution was adjusted to 5.6. To apply the excess Cu treatment, apple calli were grown on proliferation media that consisted of 300 μM CuSO_4_ for 20 days. Images of the plants and calli were taken before and after treatment.

### Detection of Cu content via micro-X-ray fluorescence (μ-XRF) microspectroscopy

The micro-X-ray fluorescence (μ-XRF) microspectroscopy experiment was performed via a 4W1B beamline system at the Beijing Synchrotron Radiation Facility (BSRF), Institute of High Energy Physics, Chinese Academy of Sciences, which runs 2.5 GeV electrons with current from 150 to 250 mA. The incident X-ray energy was monochromatized via a W/B_4_C double-multilayer-monochromator (DMM) at 15 keV and was narrowed to 50 μm in diameter by a polycapillary lens. After being treated with a 500 μM CuSO_4_ solution for 24 h, the roots and leaves of Gala 3 plants overexpressing *MdWRKY11* and those of control plants were sampled for Cu detection. Apple calli with altered levels of *MdHMA5* expression were collected after excess Cu treatment on media consisting of 300 μM CuSO_4_ for 20 days. A cryotome was used to obtain 200-mm thick root cross-sections and calli sections, and the sections were placed on Kapton tape and freeze-dried in a vacuum freeze dryer (LGJ-10B, Beijing Four-Ring Science Instrument Factory). The sample was held on a precision motor-driven stage and scanned at 60-μm intervals by two-dimensional mapping. A Si (Li) solid-state detector was used to detect XRF emission lines with a live time of 60 s. The data were processed using the PyMCA package^42,43^.

### Heterologous MdWRKY11 expression in *E. coli* and preparation of polyclonal antibodies

The coding sequence of *MdWRKY11* was amplified and inserted into a pGEX-6p-1 vector. The GST-MdWRKY11 fusion protein was then expressed and purified as described by Zheng et al.^44^. Polyclonal anti-MdWRKY11 antibodies was prepared using the method approved by the Beijing Municipal Commission of Science and Technology^44^.

### Chromatin immunoprecipitation-qPCR (ChIP-qPCR) assays

ChIP assays involving anti-MdWRKY11 polyclonal antibodies were performed as described by Zheng et al.^39^. The primers for ChIP-qPCR were designed to amplify regions in the promoter sequence of *MdHMA5* (Table S2). The experiment was performed in triplicate.

### Electrophoretic mobility shift assays (EMSAs)

EMSAs were performed using a LightShift Chemiluminescent EMSA Kit (Thermo Scientific, Waltham, MA, USA) as described by Zheng et al.^39^. The 5′ biotin-labeled *MdHMA5* promoter DNA probe containing the W-box (*proMdHMA5*-F-biotin + *proMdHMA5*-R), mutated W-box probe (*proMdHMA5* (m) -F-biotin + *proMdHMA5* (m) -R), and corresponding competitor DNA sequences (*proMdHMA5*-F + *proMdHMA5*-R) are listed in Table S2.

### Detection of gene expression in the transiently transformed apple calli

To determine the effects of *MdWRKY11* overexpression on *MdHMA5* expression, the promoter of *MdHMA5* was amplified and inserted into a pCAMBIA1301 vector to generate a *proMdHMA5::GUS* construct. Apple calli were transiently cotransformed with *Agrobacterium* EHA105 strains carrying *35S7MdWRKY11-GFP*, *proMdHMA57GUS*, and *35S7LUC*. Apple calli cotransformed with *Agrobacterium* strains carrying *35S::GFP*, *proMdHMA5::GUS*, and *35S::LUC* were used as controls. The GUS and LUC activities were determined as described by Zheng et al.^39^. The GUS:LUC activity ratio was used as the ultimate quantification of GUS activity. Each experiment was independently repeated three times.

### Phylogenetic analysis

With respect to the phylogenetic analysis of MdHMA5, previously annotated HMA5 homologs from *Arabidopsis thaliana* (AT1G63440), *Oryza sativa* (OS04G46940), *Fragaria vesca* (FvH4_5g02091), *Vitis vinifera* (XP_010651259.1), *Cucumis sativus* (CSPI05G05530), *Zea mays* (ZM2G143512), *Nicotiana attenuata* (XP_019241377.1), *Glycine max* (GM11G024400), and *Pyrus* x *bretschneideri* (XP_018506630.1) were retrieved from GenBank and aligned with MUSCLE in MEGA 7. A phylogenetic tree was then constructed using the neighbor-joining method with 1,000 bootstraps.

## Supplementary information

Revised-Supplementary Information

## References

[1] Li W, Zhang M, Shu H (2005). Distribution and fractionation of copper in soils of apple orchards. Environ. Sci. Pollut. R..

[2] Liu CS (2011). Copper toxicity and accumulation in potted seedlings of three apple rootstock species: implications for safe fruit production on copper-polluted soils. J. Plant. Nutr..

[3] Brun A, Maillet J, Hinsinger P, Pépina M (2001). Evaluation of copper availability to plants in copper-contaminated vineyard soils. Environ. Pollut..

[4] Wang QY, Liu JS, Cheng S (2015). Heavy metals in apple orchard soils and fruits and their health risks in Liaodong peninsula, Northeast China. Environ. Monit. Assess..

[5] Girotto E (2014). Copper availability assessment of cu-contaminated vineyard soils using black oat cultivation and chemical extractants. Environ. Monit. Assess..

[6] Adrees M (2015). The effect of excess copper on growth and physiology of important food crops: a review. Environ. Sci. Pollut. Res..

[7] Kan SH, Sun BY, Liu CS (2010). Toxic effects of long-term low-dose Copper (Cu) stress on apple trees in brown soils. J. Agro-Environ. Sci..

[8] De Forest DKC, Meyer JS (2015). Critical review: toxicity of diet borne metals to aquatic organisms. Crit. Rev. Env. Sci. Tec..

[9] Hippler FWR (2016). Citrus rootstocks regulate the nutritional status and antioxidant system of trees under copper stress. Environ. Exp. Bot..

[10] Leng X (2015). Transporters, chaperones, and P-type ATPases controlling grapevine copper homeostasis. Funct. Integr. Genomics.

[11] Li Q (2019). Excess copper effects on growth, uptake of water and nutrients, carbohydrates, and PSII photochemistry revealed by OJIP transients in Citrus seedlings. Environ. Sci. Pollut. Res..

[12] Wang QY, Liu JS, Hu B (2016). Integration of copper subcellular distribution and chemical forms to understand copper toxicity in apple trees. Environ. Exp. Bot..

[13] Nishizono H, Ichikawa H, Suzikp S, Ishii F (1987). The role of the root cell wall in the heavy metal tolerance of *Athyrium yokoscense*. Plant Soil.

[14] Burkhead JL, Reynolds KA, Abdel-Ghany SE, Cohu CM, Pilon M (2009). Copper homeostasis. N. Phytol..

[15] Kholodova, V. P., Ivanova, E. M., & Kuznetsov, V. V. Initial steps of copper detoxification: outside and inside of the plant cell. In *Detoxification of Heavy Metals, Soil Biology* 30 (eds. Sherameti, I. & Varma, A.) Ch. 8, 143–167 (Springer-Verlag Berlin Heidelberg, 2011).

[16] Andres-Colas N (2006). The Arabidopsis heavy metal P-type ATPase HMA5 interacts with metallochaperones and functions in copper detoxification of roots. Plant J..

[17] Cobbett CS, Hussain D, Haydon MJ (2003). Structural and functional relationships between type 1B heavy metal-transporting P-type ATPases in Arabidopsis. N. Phytol..

[18] Argüello JM (2003). Identification of ion-selectivity determinants in heavy-metal transport P 1B-type ATPases. J. Membr. Biol..

[19] Yruela I (2009). Copper in plants: acquisition, transport and interactions. Funct. Plant Biol..

[20] Kobayashi Y (2008). Amino acid polymorphisms in strictly conserved domains of a P-Type ATPase HMA5 are involved in the mechanism of copper tolerance variation in Arabidopsis. Plant Physiol..

[21] Li YB (2017). Two Silene vulgaris copper transporters residing in different cellular compartments confer copper hypertolerance by distinct mechanisms when expressed in Arabidopsis thaliana. N. Phytol..

[22] Zhang YY (2018). OsATX1 interacts with heavy metal P1B-Type ATPases and affects copper transport and distribution. Plant Physiol..

[23] Lange B (2017). Copper and cobalt accumulation in plants: a critical assessment of the current state of knowledge. N. Phytol..

[24] Deng FL, Yamaji N, Xia JX, Ma JF (2013). A member of the heavy metal P-type ATPase OsHMA5 is involved in xylem loading of copper in rice. Plant Physiol..

[25] He L (2016). Maize OXIDATIVE STRESS2 homologs enhance cadmium tolerance in Arabidopsis through activation of a putative SAM-dependent methyltransferase gene. Plant Physiol..

[26] Khare D (2017). Root avoidance of toxic metals requires the GeBP-LIKE 4 transcription factor in Arabidopsis thaliana. N. Phytol..

[27] Lin TT, Yang WN, Lu W, Wang Y, Qi XT (2017). Transcription factors PvERF15 and PvMTF-1 form a cadmium stress transcriptional pathway. Plant Physiol..

[28] Yang G (2016). Overexpression of ThVHAc1 and its potential upstream regulator, ThWRKY7, improved plant tolerance of cadmium stress. Sci. Rep..

[29] Kropat J (2005). A regulator of nutritional copper signaling in Chlamydomonas is an SBP domain protein that recognizes the GTAC core of copper response element. Proc. Natl Acad. Sci. USA.

[30] Ruzsa SM, Scandalios JG (2003). Altered Cu metabolism and differential transcription of Cu/ZnSod genes in a Cu/ZnSOD-deficient mutant of maize: evidence for a Cu-responsive transcription factor. Biochemistry.

[31] Yamasaki H, Hayashi M, Fukazawa M, Kobayashi Y, Toshiharu Shikanai T (2009). SQUAMOSA promoter-binding protein-like 7 is a central regulator for copper homeostasis in Arabidopsis. Plant Cell.

[32] Ding ZJ, Yan JY, Xu XY, Li GX, Zheng SJ (2013). WRKY46 functions as a transcriptional repressor of ALMT1, regulating aluminum-induced malate secretion in Arabidopsis. Plant J..

[33] Hong CY (2017). The role of ZmWRKY4 in regulating maize antioxidant defense under cadmium stress. Biochem. Bioph. Res. Commun..

[34] Yan JY (2016). A WRKY transcription factor regulates Fe translocation under Fe deficiency in Arabidopsis. Plant Physiol..

[35] Dai X, Wang Y, Zhang WH (2016). OsWRKY74, a WRKY transcription factor, modulates tolerance to phosphate starvation in rice. J. Exp. Bot..

[36] Jiang Y, Liang G, Yang S, Yu D (2014). Arabidopsis WRKY57 functions as a node of convergence for jasmonic acid- and auxin-mediated signaling in jasmonic acid-induced leaf senescence. Plant Cell.

[37] Li W, Wang H, Yu D (2016). Arabidopsis WRKY transcription factors WRKY12 and WRKY13 oppositely regulate flowering under short-day conditions. Mol. Plant.

[38] Iglesias I, Echeverría G, Soria Y (2008). Differences in fruit colour development, anthocyanin content, fruit quality and consumer acceptability of eight ‘Gala’ apple strains. Sci. Hortic.-Amst..

[39] Zheng XD (2018). MdWRKY9 overexpression confers intensive dwarfing in the M26 rootstock of apple by directly inhibiting brassinosteroid synthetase MdDWF4 expression. N. Phytol..

[40] Dai H (2013). Development of a seedling clone with high regeneration capacity and susceptibility to Agrobacterium in apple. Sci. Hortic.-Amst..

[41] An XH (2015). MdMYB9 and MdMYB11 are involved in the regulation of the JA-induced biosynthesis of anthocyanin and proanthocyanidin in apples. Plant Cell Physio..

[42] Kim SA (2006). Localization of iron in Arabidopsis seed requires the vacuolar membrane transporter VIT1. Science.

[43] Solé VA, Papillon E, Cotte M, Walter P, Susini J (2007). A multiplatform code for the analysis of energy-dispersive X-ray fluorescence spectra. Spectrochim. Acta B.

[44] Zheng XD (2017). Chloroplastic biosynthesis of melatonin and its involvement in protection of plants from salt stress. Sci. Rep..

