# Supplementary material for: MdWRKY11 improves copper tolerance by directly promoting the expression of the copper transporter gene MdHMA5
Source: Hortic Res. 2020 Jul 1;7:105. doi: 10.1038/s41438-020-0326-0 (PMC7327004; doi:10.1038/s41438-020-0326-0)
Supplement: Supplementary file 1 — Revised-Supplementary Information [file 41438_2020_326_MOESM1_ESM.docx]

**Supplementary Information**

**MdWRKY11 improves copper tolerance by directly promoting the expression of the copper transporter gene *MdHMA5***

**This file includes:**

Supplementary Fig. S1 to S5

Supplementary Table S1 to S2

Supplementary Fig. S1:


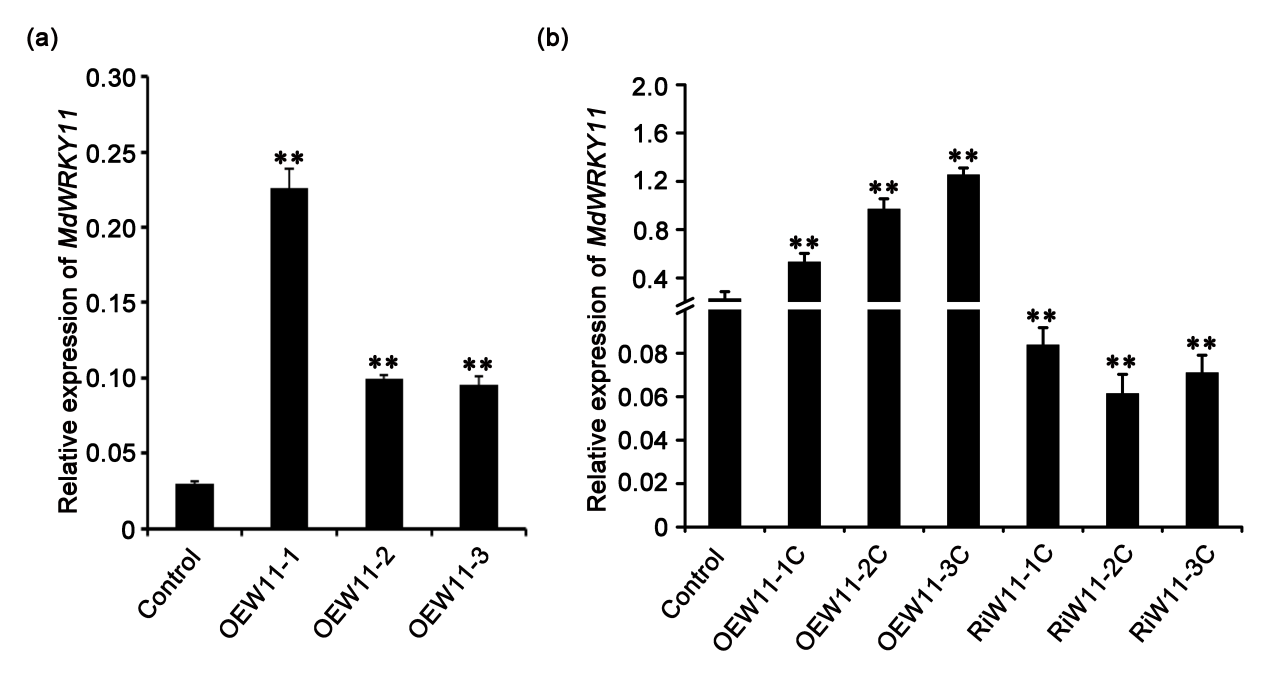


Fig. S1 Relative expression of *MdWRKY11* in transgenic apple plants overexpressing *MdWRKY11* and transgenic calli with increased or reduced *MdWRKY11* expression. (a) Relative expression of *MdWRKY11* in transgenic apple plants overexpressing MdWRKY11 (OEW11-1, OEW11-2, and OEW11-3) and control untransformed plants. (b) Relative expression of *MdWRKY11* in transgenic *MdWRKY11-*overexpressing (OEW11-1C, OEW11-2C, and OEW11-3C) or *MdWRKY11* RNAi (RiW11-1C, RiW11-2C, and RiW11-3C) calli and untransformed control calli. Data are means ± SD of triplicate experiments. Asterisks indicate the values are significantly different from that of the control (Student’s *t*-test): **, *P* < 0.01.

Supplementary Fig. S2:


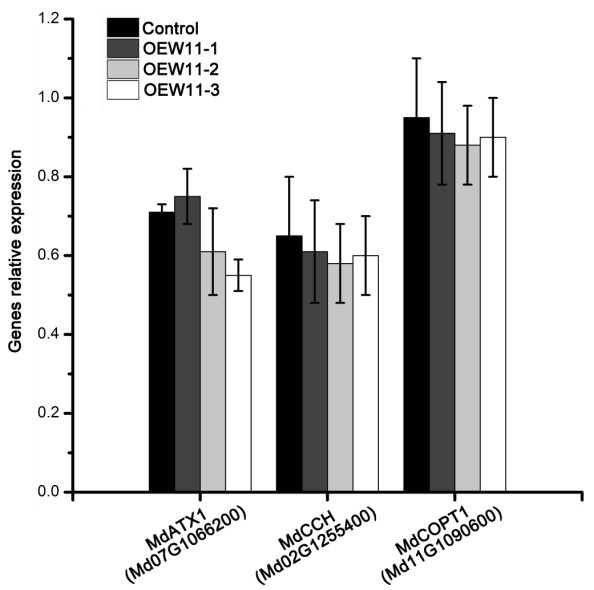


Fig. S2 Relative expression of MdATX1, MdCCH and MdCOPT1 in transgenic plants overexpressing *MdWRKY11* and control plants. Data are means ± SD of triplicate experiments.

Supplementary Fig. S3:


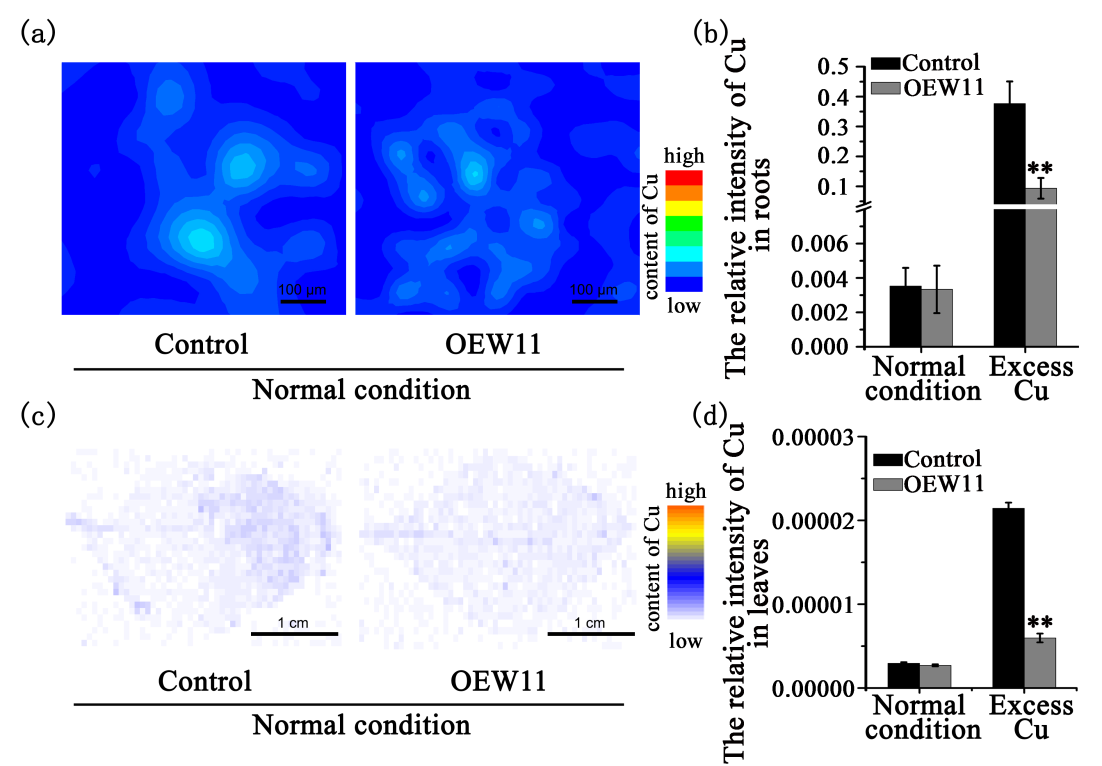


Fig. S3 Cu level and distribution shown by μ-XRF elemental mapping in roots and leaves under normal conditions. (a) Cu level and distribution in roots under normal conditions. (b) Relative intensity of Cu in roots under normal conditions and excess Cu. (c) Cu level and distribution in leaves under normal conditions. (d) Relative intensity of Cu in leaves under normal conditions and excess Cu.

Supplementary Fig. S4:


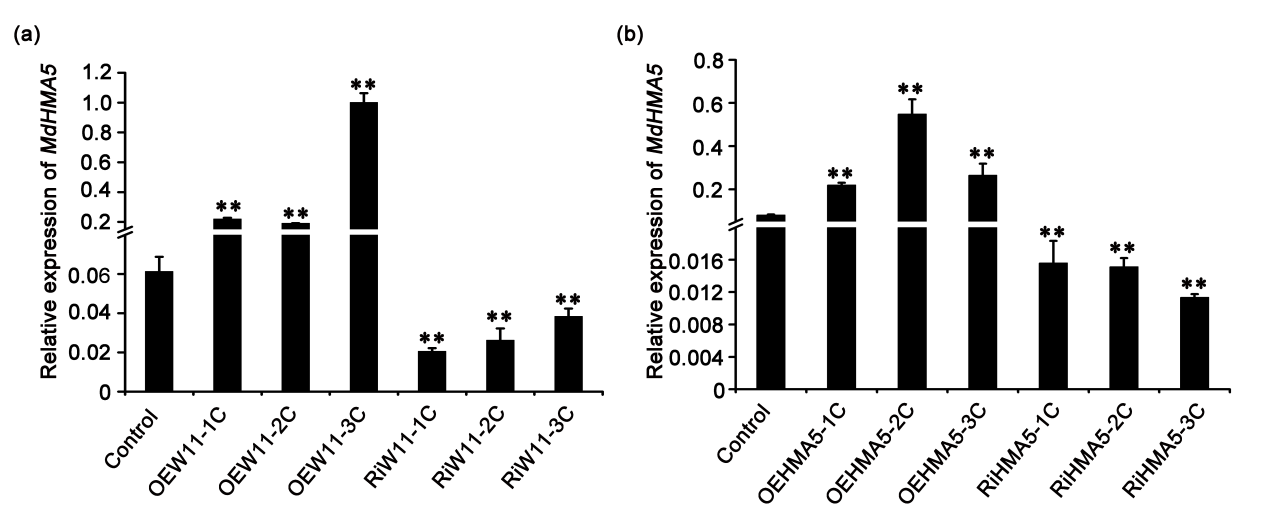


Fig. S4 Relative expression of MdHMA5 in transgenic calli with manipulated expression of MdWRKY11 or MdHMA5. (a) Relative expression of MdHMA5 in transgenic callus lines with overexpressed MdWRKY11 (OEW11-1C, OEW11-2C, and OEW11-3C) or RNA interfered MdWRKY11 (RiW11-1C, RiW11-2C, and RiW11-1C) and control calli. (b) Relative expression of MdHMA5 in transgenic calli overexpressing MdHMA5- (OEHMA5-1C, OEHMA5-2C, and OEHMA5-3C) or MdHMA5 RNAi (RiHMA5-1C, RiHMA5-2C, and RiHMA5-3C) and untransformed control calli. Data are means ± SD of triplicate experiments. Asterisks indicate the values are significantly different from that of the control (Student’s t-test): **, *P* < 0.01.

Supplementary Fig. S5:


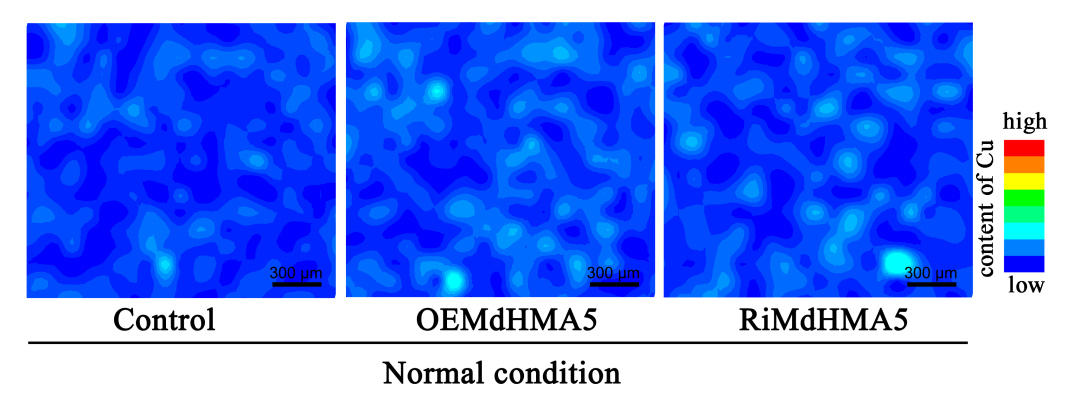


Fig. S5 Cu level shown in μ-XRF elemental maps of transgenic apple calli with increased or reduced *MdHMA5* expression and control calli under normal conditions.

Supplementary Table S1 Primers used for qPCR

| Gene name | Accession number | Forward primer | Reverse primer |
| --- | --- | --- | --- |
| *MdWRKY11* | Md15G1054000 | AGCAGCCAACATGGATTGTCTA | AAGGCACCCTTCAACTGAGCAC |
| *MdHMA5* | Md03G1182600 | CGATAAACATTACAAGCATCAT | TCTGCGTTCAAAACCCGAACAA |
| *MdActin* | Md01G1001600 | CTTCAATGTGCCTGCCATGTAT | AATTTCCCGTTCAGCAGTAGT |

Note: The specific primers were designed according to the gene sequences using Primer 5 software.

Supplementary Table S2 Primers used for vector construction, ChIP-qPCR, and EMSA

| Primer name | Forward primer | Reverse primer | Vector |
| --- | --- | --- | --- |
| pBI121-MdWRKY11 | TCTAGAATGTCTGGTGGCAACTT | CCCGGGGAGAGGCCTG | pBI121 |
| GFP-MdWRKY11 | TTAATTAAATGTCTGGTGGCAACTT | GGCGCGCCAGAGGCCT | pMDC83 |
| pBD-MdWRKY11 | CCATGGAGATGTCTGGTGGCAACTT | GTCGACGGAGGCCTGGATGGTTAT | pGBKT7 |
| GST-MdWRKY11 | GTCGACATGTCTGGTGGCAACTT | CTCGAGTTAGAGGCCTGGATGGT | pGEX-6P-1 |
| RNAi-MdWRKY11  first | TCTAGAATGTCTGGTGGCAACTTCAG | GGATCCTCAACCTCTGATTTTGTTTTG | pZH01 |
| RNAi-MdWRKY11  second | GAGCTCATGTCTGGTGGCAACTTCAG | GGTACCTCAACCTCTGATTTTGTTTTG | pZH01 |
| GFP-MdHMA5 | TTAATTAAATGTGTGTGTTGGTTTCCATT | GGCGCGCCAATCTGCGT | pMDC83 |
| RNAi-MdHMA5  first | TCTAGATACTGGTGATAACTGGGCTA | GTCGACCGTTCAAAACCAGAACAAA | pZH01 |
| RNAi-MdHMA5  second | GAGCTCTACTGGTGATAACTGGGCTA | GGTACCCGTTCAAAACCAGAACAAA | pZH01 |
| GUS-proMdHMA5 | TCTAGAAGTCCCCGCAGCG | AGATCTTCGAGCTAGATTATTATGTTCTGAAA | pCAMBIA1301 |
| For ChIP-qPCR of  MdHMA5-P1 | TTATGCACTTCATTCCTGACCAGT | AAAGGACAAAACAATTAATAGCCTTTTC |  |
| For ChIP-qPCR of  MdHMA5-P2 | AGACACCTCATTGTTATTATGCTATTG | TCGAGCTAGATTATTATGTTCTGAAATG |  |
| For EMSA of  *proMdHMA5*-F-biotin | AGGTTGGTCCTGAGGTTGACAAGTACATTTCAGAACATAAT | | 5′ biotin-labeled DNA probe |
| For EMSA of  *proMdHMA5*-R | ATTATGTTCTGAAATGTACTTGTCAACCTCAGGACCAACCT | |  |
| For EMSA of  *proMdHMA5*(m)-F-biotin | AGGTTGGTCCTGAGGTTAACAAGTACATTTCAGAACATAAT | | mutated probe |
| For EMSA of  *proMdHMA5*(m)-R | ATTATGTTCTGAAATGTACTTGTTAACCTCAGGACCAACCT | |  |
| For EMSA of  *proMdHMA5*-F | AGGTTGGTCCTGAGGTTGACAAGTACATTTCAGAACATAAT | | competitor DNA sequence |
| For EMSA of  *proMdHMA5*-R | ATTATGTTCTGAAATGTACTTGTCAACCTCAGGACCAACCT | |  |
